# Supplementary material for: Are carbon emissions trading and green financial instruments synergistic? -Comprehensive quantitative research based on content analysis
Source: PLoS One. 2024 Mar 7;19(3):e0298601. doi: 10.1371/journal.pone.0298601 (PMC10919601; doi:10.1371/journal.pone.0298601)
Supplement: S1 Data — (ZIP) [file pone.0298601.s001.zip › Data/地级市Z_F工作报告.zip!/╦─┤¿/╦∞─■╩╨/└┤╫╘╣1⁄2╓┌║┼í╛┬φ┐╦╩2╛▌═°í┐_2016.docx]

各位代表：

现在，我代表市人民政府向大会报告工作，请予审查，并请市政协委员提出意见。

　　2015年主要工作及"十二五"发展成就

2015年，是经济下行压力持续加大、多重挑战交织叠加的一年。在市委的坚强领导下，在市人大、市政协的监督支持下，我们紧紧依靠全市人民，沉着应对挑战、主动破解难题，深入推进"六大兴市计划"，主要经济指标提速升位。地区生产总值增长13.2%，地方一般公共预算收入增长23.6%，规模以上工业增加值增长18.0%，服务业增加值增长12.0%，民营经济增加值增长12.7%，五项指标增速均居全省第一位，建市30年来尚属首次。全社会固定资产投资突破千亿大关，社会消费品零售总额、税收收入等指标增速位居全省前列。

　　一年来，困难比预想的大，效果比预想的好。我们顶住压力、迎难而上，主要做了以下工作。

　　一、定向发力，调优结构强产业

　　大力发展新型工业。新增规模以上工业企业43家，四大成长型工业产值增速比全市工业平均水平高12.9个百分点。与中石油成功合资组建天然气净化有限公司，实现年产值66亿元、税收5.5亿元。江淮汽车西南基地全面建成投产，带动福多纳等10余户配套企业入驻。洪芯微电子、上特科技等电子企业投产，推动由"切芯片"向"造芯片"跨越。加快传统企业兼并重组，沱牌舍得完成股权转让及增资扩股，成为全省酒类企业混合所有制改革第一宗成功案例；华润锦华引进创维数字建设创维西部产业园；盛马化工引入合作伙伴推进战略重组。实施之江新材料等435个技改项目，技改投资增长16.7%。全面落实结构性减税和普遍性降费政策，减轻企业负担4.9亿元。建立5亿元应急转贷资金池，为企业提供转贷资金16.2亿元。

　　培育壮大服务业。把服务业作为调结构的主攻方向，远成西部物流园开园，威斯腾物流园铁路专线启动铺轨，物流港蝉联全国优秀物流园区。7家大型电商平台投入运营，电子商务交易额增长25.7%。全力推进26个重点旅游项目建设，成功举办第八届中国（遂宁）观音文化旅游节暨世界荷花博览园开园活动，全市实现旅游收入251亿元、增长25.0%。创新推出"遂心税易贷""助保贷"等金融产品，金融机构存贷款余额分别增长20.0%、20.5%；10家企业在区域股权交易市场挂牌，全市实现资本市场融资80亿元。加强科技服务，新增国家高新技术企业14家，专利申请量增长17.9%。

　　积极发展现代农业。实现粮食总产159.6万吨，优质水果、绿色蔬菜、经济林木、优质畜禽等特色效益农业加快发展，"遂宁鲜"农产品区域公用品牌走向市场。完成五大现代农业园区大环线规划，建成核心区15.5万亩。积极培育新型农业经营主体，新增市级以上龙头企业13家、市级示范农民合作组织18家、家庭农场233家。遂宁农业科技园区被科技部认定为国家农业科技园区。加强农业综合生产能力建设，新建高标准基本农田41万亩，整治病险水库15座，新增农村沼气用户7511户。

　　二、精准施策，挖潜借力添后劲

　　突出改革创新增活力。深化简政放权，全面取消非行政许可审批，"三证合一"、集群注册等商事制度改革扎实推进，注册企业增加3763户、增长27.7%。深化农村产权、国有企业、财政金融等改革，成立全省首个市级农村产权交易服务中心，全市实现交易189宗、10.1万亩，农村产权抵押贷款达1.7亿元；市属国有企业从49户整合到23户；财政"四本预算"体系全面建立；射洪农信社改制为农商行，遂宁市商业银行获批在成都设立分支机构。大力推进"大众创业、万众创新"，在全省率先出台"双创"23条、就业创业27条措施，设立创新创业引导及担保基金，建成创业及科技孵化园区17个，新增回乡创业人员3660人、大学生创新创业1421人。

　　突出投资消费强内需。深入实施"6382"项目计划，完成全社会固定资产投资1011.6亿元、增长10.7%。遂广、遂西高速建成通车，观音湖隧道投入使用，中环线、柳树电航工程建设有序推进，三仙湖水库、祥凤寨水库、群英水库实现预期目标，四川蓬船灌区环评报告通过环保部评审，唐家渡电航工程进入施工招标阶段。创新项目投融资方式，试点推行PPP模式，争取国家专项建设基金22亿元、地方政府债券转贷资金和在建项目后续融资72.3亿元，政策性银行低息长周期贷款余额达104亿元。大力激活城乡消费，社会消费品零售总额增长13.4%。积极参加"川货全国行"等省级市场拓展活动，举办川渝（遂宁）精品粮油展示展销会等市级促销活动100余场次，消费增速连续10个月高于投资。政府购买公共服务达60项、3.14亿元，分别增长76%和56%。全面落实房地产新政，商品房销售面积增长10.8%。

　　突出开放合作拓空间。强化高位招商、小分队招商，积极参加中国西部（四川）进出口商品展暨国际投资合作洽谈会、科博会、农博会等，成功举办遂宁市重点项目上海推介会、四川暨遂宁优质农产品北京展销周等活动，引进协鑫集团、杭萧钢构、中科光芯、湖北车桥等10亿元以上项目20个，到位市外资金422.5亿元。西部国际技术合作产业园开创了政企合作建园新模式，经开区创建成为国家新型工业化产业示范基地。携手珠海市、黔南州、广东物流行业协会共建川贵广—南亚国际物流大通道。积极发展外向型经济，实现进出口总额6.3亿美元，实际利用外资7261万美元。

　　三、协调推进，统筹城乡优环境

　　城市建设优化提升。突出规划引领，城市新区基本实现控规全覆盖。成为国家海绵城市建设试点城市，出台国内首个海绵城市专项规划，完成试点项目31个。公园路高架桥、开善东路改造及风貌整治等54个城镇环境提升项目全面竣工，"五个二"建设项目有序推进。加强城市管理，实施地理空间信息平台、电子警察等智慧城市建设项目6个，数字城管系统综合测评全省第一。深入推进多城联创，国家环保模范城市创建工作通过环保部西南督查中心预督查，创建全国文明城市首轮国检排名靠前。

　　镇村建设有序推进。实行乡村规划师和规划督察员制度，在全省率先实现幸福美丽新村规划全覆盖。深入开展"绿色城镇建设行动"，建成拦江、沱牌、龙凤3个绿色小城镇。扩权强镇试点镇达到12个。围绕基础设施、新型村庄、产业发展、公共服务和社会管理综合配套，启动幸福美丽新村建设244个。加速推进乡村道路建设和改造，建成通村公路272.4公里，改善农村公路406.8公里。实施城乡建设用地增减挂钩项目21个，争取周转指标2259.9亩。

　　城乡环境上档升级。全面贯彻新环保法，查办环境违法案件40件。强力整治大气污染，PM10浓度值下降6.9%。加强境内涪江流域环境综合治理、农村环境连片治理等工作，涪江老池出境断面和渠河饮用水源水质达标率达100%。初步划定国家级生态保护红线，成为国家生态文明先行示范区建设地区。治理水土流失131.2平方公里，完成营林造林16.2万亩，森林覆盖率达39.01%。

　　四、改善民生，共建共享促和谐

　　民生实事全面完成。民生支出达126.2亿元，占地方一般公共预算支出的69%，10项民生工程、21件民生大事圆满完成。城乡居民人均可支配收入分别增长8.1%、9.8%；精准减贫4.4万人；城镇登记失业率控制在4.2%以内；解决17.9万农村人口饮水不安全问题；新开工城镇保障性住房16219套；改造农村危旧房2572套；建成征地拆迁农民安置房193万平方米；解决失地农民养老保险历史欠账2.6亿元；城乡低保月人均补差分别提高38.5元、28.5元；市级残疾人康复中心动工建设。

　　公共服务提质扩面。组建10个"名校 弱校"区域性教育集团，高考录取率创历史新高，乡镇公办幼儿园实现全覆盖。建立健全分级诊疗及双向转诊制度，城乡居民住院政策范围内报销比例达75%。打通城乡居民与企业职工养老保险转移衔接通道，城乡居民基本养老保险参保人数达94万。遂宁杂技团《青花瓷》亮相2015年央视春晚，央视《寻宝》节目走进遂宁，大型川剧《燕归》在全市巡演。启动300个农民体育健身工程项目建设。

　　社会保持和谐稳定。依法稳妥处置民间融资风险，追缴冻结资产16亿元，风险总体可控。深化安全生产大检查，推进道路交通、食品药品等领域专项整治，安全生产保持平稳态势。加强社会治安综合治理，布建天网视频监控点831个，设置街面屯警点26个，命案破案率达100%，农村"雪亮工程"在全省推广。创新建立社会稳定第三方风险评估机制，引导群众理性反映诉求，越级上访和集访次数分别下降19.1%、4.6%。恢复设立市应急委，全市有效处置突发事件380起。"12345"政府服务热线受理群众诉求47.9万件，办理满意率达92.3%。"六五"普法圆满完成。国防动员、人防、民兵预备役、双拥、民族宗教、保密、妇女儿童、地方志、档案等工作扎实推进。

　　各位代表，2015年各项目标任务的顺利完成，为"十二五"发展画上了圆满的句号。回首"十二五"，全市广大干部群众励精图治、砥砺奋进，开创了经济社会发展新局面。

　　过去五年，我们保持专注发展定力，多措并举稳增长，跃上了追赶跨越新起点。我们牢牢抓住发展第一要务，地区生产总值相继跨越5个百亿台阶，总量翻了近一番；地方一般公共预算收入从17.8亿元增加到49.3亿元，是2010年的近3倍。县域经济竞相发展，射洪经济总量近300亿元，大英、蓬溪、安居、经开区、船山经济总量先后跨百亿元，经开区升格为国家级经济技术开发区。

　　过去五年，我们坚定转型升级信心，主动作为调结构，走出了绿色发展新路子。我们牢固树立转型才能更好发展的理念，积极探索绿色发展新模式，成功承办两届"绿色经济·遂宁会议"，成为全省乃至全国绿色发展典范。产业结构由2010年的21.5：51.8：26.7调整为15.5：56.1：28.4，万元GDP能耗下降24%。新产业、新产品、新业态加快孕育成长，成为全省电子信息产业配套基地、全国优秀物流城市。新型城镇化步伐加快，构建了"一核两带三片"城镇体系，常住人口城镇化率提高7.7个百分点。中心城区人口由不足50万增加到近80万，建成区面积由52平方公里拓展到78平方公里，形成了"一城两区五组团"发展格局。成功跻身全球绿色城市、国际花园城市、中国十佳宜居城市，获得城市名片近20张，成为西部地区最美丽的二线城市之一。成功承办省第十二届运动会、省第八届老运会开幕式、省第八届残运会暨第三届特奥会等大型赛事。

　　过去五年，我们深入实施开放战略，蹄疾步稳促改革，催生了经济增长新动能。我们坚持谋定而后动，有序实施15个方面233项改革，承担中省改革试点示范任务52项。四种方式托管土地、绿色发展等改革试点经验被中央改革办推广。纵深推进开放合作，遂宁海关开关、公用型保税仓库建成投运，告别了本地企业"外地报关"历史。五年引进市外资金1926.8亿元。全市建成"1环8射"高速路网、"3向7线"铁路枢纽，形成至成渝及周边城市90分钟交通圈，在全省的战略地位更加凸显。

　　过去五年，我们坚持为民服务宗旨，全心全意惠民生，营造了和谐安康新环境。我们坚持将财力向民生倾斜，民生投入达494.2亿元，占地方一般公共预算支出的68.08%，城乡居民人均可支配收入分别达到25012元、11379元，是五年前的1.8倍和2.3倍。持续提升民生保障水平，在全省率先统筹城乡居民基本养老保险和医疗保险，率先实行大病医疗保险；全市五年减贫46万人，农村绝对贫困发生率从19.5%下降到3.8%。义务教育"三免一补"实现全覆盖，学前三年毛入园率从67%提高到85%。县级公立医院全部取消以药补医，医疗卫生水平明显提高。新建和改建市体育中心等体育场馆14个。市县乡村四级公共文化设施基本建成并全部免费开放。

　　各位代表，"十二五"取得的成绩来之不易，是省委、省政府深切关怀、坚强领导的结果，是市委科学谋划、统揽全局的结果，是市人大和市政协广泛监督、大力支持的结果，是全市人民团结一致、奋力拼搏的结果。在此，我代表市人民政府，向关心支持遂宁发展的各界人士和全市人民，表示最衷心的感谢并致以最崇高的敬意！

　　回顾"十二五"，我们深刻认识到，做好政府工作，必须牢牢把握稳中求进这个"总基调"，为未来发展打下更加坚实的基础；必须牢牢把握改革开放这个"总动力"，在不断拓宽发展空间中打开新局面；必须牢牢把握转型升级这个"总路径"，走向更高质量更高水平的发展道路；必须牢牢把握群众满意这个"总目标"，让老百姓过上更加美好的生活。

　　我们也清醒地认识到，在前进道路上还面临不少困难和问题。我市与全国、全省的发展差距仍然较大，人均生产总值仅为全国的56.7%、全省的75.7%；长期积累的结构性矛盾比较突出，产业转型升级任重道远；去产能、去库存、去杠杆、降成本、补短板任务繁重；还有10.6万人没有脱贫，扶贫攻坚任务艰巨；社会和谐稳定依然面临不少压力；少数政府工作人员服务意识差，不作为、乱作为等现象还没有彻底扭转。对这些问题，我们一定以对人民高度负责的态度，采取有力措施，努力加以解决，决不辜负人民群众的信任和期望！

　　"十三五"发展的主要考虑

　　"十三五"是全面建成小康社会的决战决胜阶段。按照党的十八届五中全会、省委十届七次全会、市委六届十次全会精神和市委关于"十三五"规划编制的建议，市政府组织编制了《遂宁市国民经济和社会发展第十三个五年规划纲要（草案）》，已印发大会审议。这里，重点从三个方面简要说明。

　　关于发展形势。今后五年，我们面临的国内外发展形势依然错综复杂。从国际看，世界经济仍处在深度调整期，存在许多不确定因素。从国内看，在以速度换挡、结构优化、动力转换为主要特征的经济新常态下，调结构、转方式、防风险的任务依然繁重。但从长远看，经济长期向好的基本面没有改变，仍处于大有作为的重要战略机遇期。一方面，国家大力实施"一带一路"战略、长江经济带建设，全省深入实施多点多极支撑发展战略，全面推进创新改革试验，为我们提供了重大机遇。另一方面，建市30多年来，历届班子一届接着一届干，在产业发展、城乡建设、民生工作等方面打下了坚实的基础，遂宁迈入了蓄势突破的发展新阶段：经济发展由增速优先向量质并重转变；产业结构由传统模式向更高层次转变；增长动力由投资拉动向多元支撑转变；县域经济由单体突破向协调发展转变；城市建设由规模扩张向内涵提升转变；人民生活由总体小康向全面小康转变。"六个转变"既是新常态下遂宁发展所积蓄的重要动能和积极因素，也是我们必须肩负的历史使命和需要完成的重大任务。我们将始终保持专注发展定力，从容应对挑战，奋力攻坚克难，闯过转型发展的重要历史关口，全力建设大而美、富而强、优而雅的新遂宁。

　　关于目标定位。围绕"决战决胜全面小康，建设绿色经济强市"发展主题，把遂宁建成四川次级综合交通枢纽、西部重要现代产业基地、以"养心"文化为特色的现代生态花园城市，成为成渝经济区重要的区域性中心城市。预期目标是：经济发展保持高于全省的增长速度，到"十三五"末实现地区生产总值1500亿元，比2010年翻一番以上；转型升级步伐加快，产业迈向中高端水平；居民收入较快增长，城乡差距进一步缩小；率先完成脱贫攻坚任务，基本公共服务均等化水平稳步提高；绿色经济基础更加坚实，生态环境质量持续改善；开放型经济新体制基本形成；法治政府基本建成。

　　围绕目标定位，我们规划储备了1292个重点项目，总投资达10589亿元。将重点推动与成都经济区同城化路网建设，加快建成"1环9射"高速路网、"5向11线"铁路枢纽和遂宁通用机场，推进涪江航道建设，构建立体的综合交通网络；重点推动产业转型升级，推进园区"141"工程和"五大旅游度假区"、五大现代农业园区等重点项目，建成成渝经济区重要的"六基地一中心一目的地"；重点推动城乡建设上档升级，大力实施海绵城市、河东新区二期、老城区景观提升等项目，中心城区人口达到100万、建成区面积达到100平方公里，建成生态山水城、现代花园城、观音文化城，打造集约高效、品位高雅、活力迸发、宜居宜业、文明和谐的中国名城。这些美好蓝图既充分尊重了全市人民发展愿望，也充分考虑了宏观环境和支撑条件，通过我们的共同努力是能够实现的。

　　关于发展路径。做好今后五年工作，我们将围绕"五位一体"总体布局和"四个全面"战略布局，深入贯彻省委、省政府和市委的重大部署，坚持科学发展、绿色发展、创新发展、跨越发展总体取向，深入实施统筹城乡、"三化"联动发展战略，大力推进"六大兴市计划"，发挥优势，干出特色，努力在全面建成小康社会中走在全省前列。

　　根据这一总体要求，围绕"五大发展理念"，走出符合遂宁实际的创新、绿色、协调、开放、共享发展之路。一是围绕解决发展动力转换问题，着力创新驱动，打造国家创新型城市。突出在提高科技创新能力、发挥企业创新主体作用、实施人才强市战略、营造良好创新环境等方面发力，打造经济增长新引擎。二是围绕解决人与自然和谐问题，着力绿色转型，建设绿色经济强市。突出在推行绿色生产方式、构筑绿色产业体系、全域建设生态田园市、促进资源节约集约利用等方面发力，打造绿色发展新优势。三是围绕解决不平衡问题，着力统筹协调，优化发展布局。突出在次级综合交通枢纽建设、推进城乡统筹、建设智慧城市、提高社会文明程度等方面发力，形成均衡协调发展新格局。四是围绕解决深层次矛盾及与外部发展联动问题，着力改革开放，提升区域战略地位。突出在重点领域改革特别是供给侧结构性改革、投资促进、区域交流、与成都平原城市群同城化发展等方面发力，培育区域竞争新优势。五是围绕解决社会公平正义问题，着力共建共享，提高群众幸福指数。突出在脱贫攻坚、提高城乡居民收入、促进创新创业、提升教育质量、推进健康遂宁建设、完善社会保障和现代公共文化服务体系等方面发力，促进人民群众生活水平再上新台阶。

　　2016年重点工作

　　2016年，是"十三五"开局之年，也是经济总量跨进"千亿俱乐部"的决胜之年。今年政府工作的基本思路是：深入贯彻省委、省政府和市委一系列新理念新部署，主动适应把握引领经济发展新常态，始终保持专注发展、转型发展战略定力，坚持稳中求进工作总基调，深入实施"六大兴市计划"，以全面创新改革为牵引，以供给侧结构性改革为重点，统筹推进稳增长、调结构、惠民生、防风险等各方面工作，努力实现"十三五"发展良好开局。

　　综合考虑各方面因素，今年全市经济社会发展主要预期目标是：地区生产总值增长10%；地方一般公共预算收入增长12%；城乡居民人均可支配收入分别增长9%、10.5%。实现这些目标，重点抓好以下五个方面工作。

　　一、把创新发展作为核心动力，打造转型升级新引擎

　　纵深实施创新驱动战略。抢抓四川加快建成国家创新驱动发展先行省的机遇，努力探索区域创新发展新机制。充分发挥企业创新主体作用，建立健全政产学研用协同创新体系，支持天齐锂业、英创力等企业建立省级以上研发机构。深入推进国家知识产权试点城市工作。大力促进"大众创业、万众创新"，打造创新创业新平台，新建10个以上特色创新创业基地；实施遂州英才招揽行动，全面落实人才强市十条措施，选派300名管理、技术人才进行深造，引进20名产业领军型人才，鼓励科技人员离岗创业和兼职取酬；实施创业遂州行动，积极鼓励遂商返乡创业，落实大学生就业促进和创业引领计划，大力发展众创、众包、众扶、众筹等新模式。

　　突出抓好供给侧结构性改革。因企施策去产能，坚持多兼并重组、少破产清算，引导停产半停产企业走技改重组之路，稳妥有序处置"僵尸企业"。多管齐下降成本，调减市级统筹社保险种缴费率，降低人工成本；清理规范涉企收费，启动"规上企业改制三年行动计划"，加强政府"增信帮扶"，支持企业直接融资，降低财务成本；鼓励工业企业和商贸企业剥离物流业务，大力发展第三方物流，降低物流成本。多措并举去库存，进一步落实房地产新政，推动棚改安置房、公租房和租赁市场与存量商品房市场有效衔接，建立购租并举的住房制度；引导企业适当降低商品房价格。稳妥有序去杠杆，积极争取地方政府债券转贷资金，推动政府融资平台市场化转型，确保政府债务率控制在100%以内；规范民间投融资行为，引导民间资金"脱虚向实"。

　　深入推进重点领域改革。深化商事制度改革，有序实施市场准入负面清单制度，把"放、管、服"改革引向深入。深化农业农村改革，推广土地流转收益保证贷款、集体资产股份制改革等试点经验。深化财税体制改革，健全完善财政税收体系；转变财政支持产业发展方式，健全投资引导基金体系；深入推进PPP模式，撬动更多社会资本投入项目建设。深化金融改革创新，加快遂州农村信用联社、蓬溪农村信用联社改制步伐。深化国有企业改革，完善国资三级构架模式监管体系，稳妥推进混合所有制改革。深化户籍制度改革，建立和落实转移人口与建设用地、建设资金、转移支付"三挂钩"机制。

　　二、把绿色发展作为战略品牌，推动产业迈向中高端

　　加快工业转型升级。启动《中国制造2025遂宁行动计划》，推动制造业与"互联网 "融合发展，提升"遂宁造"影响力。深入实施大企业培育计划，支持中小微企业"专精特新"发展，新增规模以上工业企业40户，规模以上工业增加值增长12%。培育壮大新兴产业，加快安居天然气产业园建设，力争磨溪净化厂新增产值40亿元；延伸电子信息产业链条，推进创维西部产业园建设，促进广义微电子、威骏、盛利兴等电子信息项目投产；提升机电与装备制造产业，支持江淮集团四类整车生产资质落户遂宁，推动云内动力等10余个配套项目建设；壮大新材料产业，促进之江新材料、四川能宝等项目投运达效，天齐锂业、行盛玻璃进一步释放产能。大力提升传统产业，支持沱牌舍得、高金食品向大健康、大消费领域发展；加快建设盛马化工重油升级项目；推动锦华纺织"退城入园"，实施佐鲁银华、南华纺织等企业技改，鼓励纺织企业研发推广具有自主品牌的纺织服装产品。实施能源和水资源消耗、建设用地等总量和强度双控行动，单位工业增加值能耗下降4%。

　　提速发展现代服务业。积极对接全省"三百示范工程"，大力发展现代物流、电子商务、文化旅游、健康养老、检验检测等新型服务业，服务业增加值增长14%。推动远成西部物流园全面营运，威斯腾物流园实现200万吨运能。实施"全企入网、全民触网、电商示范"三大工程，推进电商进农村、进社区，建成1个省级电子商务集聚区，电子商务交易额增长30%。扎实推进"五大旅游度假区"建设，抓好仁里古镇、浪漫地中海等10个重点旅游项目，力争新创建2个国家4A级景区；支持河东新区观音文化产业园创建国家级文化产业示范基地；办好第九届中国（遂宁）观音文化旅游节、第三届涪江文化艺术节，旅游收入突破300亿元。加快建设医疗健康城，推行医养结合新模式，支持社会力量开办养老机构，新增民办养老机构床位2300张。

　　加快推动农业现代化。深入实施"粮安工程"，严守耕地红线，粮食产量达到159万吨以上。大力推广"遂宁鲜"农产品区域公用品牌，壮大畜禽、果蔬、中药材、经济林木等优势产业。大力发展农产品加工业，建立营销网络平台，促进农村一二三产业融合发展，力争创建全省第三轮现代农业重点县（区）3个以上。启动现代农业园区大环线建设，积极培育新型农业经营主体，完善利益联结机制。完成12座病险水库整治，加强农村小微水利工程建设，新建10万亩高标准基本农田。加大农业科技推广力度，健全农业社会化服务体系。加强气象现代化建设，增强自然灾害防御能力。做好第三次全国农业普查。

　　强化生态环境保护。持续推进长江上游生态屏障建设，实施好新一轮国土绿化工程，抓好天然林保护、退耕还林、森林抚育等重点工程，森林覆盖率达到39.2%。推进城市"蓝线""绿线"划定和管控工作，加快东西山森林公园、河东养生谷花卉公园建设，保护好自然山水园林。推进306个海绵城市试点项目建设。实施城乡环境综合整治"五大工程"，推进涪江、琼江、郪江等中小流域环境综合治理，加强明月河、联盟河等城镇水体整治；突出"减排、压煤、抑尘、治车、控秸"，深入开展大气污染治理。抓好"绿色城镇·幸福生活"示范工程，办好2016"绿色经济·遂宁会议"。加快国家生态文明先行示范区建设，积极争创全国文明城市、国家环保模范城市、全国水生态文明城市、国家节水型城市、国家低碳城市试点。做好国家卫生城市复评迎检工作。

　　三、把协调发展作为基本要求，提高全域发展平衡性

　　着力投资消费协调拉动。以全省"项目年"为契机，大力实施"6348"项目计划，稳住基础投资，加强产业投资，激活民间投资，完成全社会固定资产投资1123亿元、增长11%。突出抓好工业投资，加快喜之郎、隆鑫科技保健机器人等60个重点工业项目建设，完成工业投资390亿元；推动四川蓬船灌区、唐家渡电航工程、机场迁建主体工程等13个重大项目开工；除凤台大桥外，中环线其余路段基本建成通车。积极发挥新消费的引领作用，大力培育文化、旅游、信息、服务等消费热点，引导批零住餐等传统消费线上线下融合，新增限上商贸企业50家，确保社会消费品零售总额增长12.5%。加快专业市场、批发市场、农贸市场改造升级，推进远成物流主题园建设。积极参加省级市场拓展活动，筹办好第二届国际口岸经贸物流合作大会暨成都航空口岸开放合作大会、第三届中国国际物流发展大会暨第四届中国（四川）国际物流博览会、西部金穗粮油展等品牌展会。

　　着力城乡建设协调推进。坚持以"五个统筹"推进新型城镇化，常住人口城镇化率提高1.5个百分点。推进市县"多规合一"，建立规划违法违纪约谈制度和党政主要领导离任审计制度，严格规划管控。开展"城市基础设施建设年行动"，加快停车场规划建设，推进滨江中路拓宽、通善大桥整治等缓堵保畅工程；推动新型建筑工业化产品运用，力争完成可再生能源建筑应用示范面积185万平方米；加强城市地下综合管廊改造建设，治理城市内涝；支持各县（区）有序拓展城市空间，推进旧城改造，力争棚改货币化安置比例超过50%；启动遂宁——安居快捷通道扩宽工程前期工作，加快遂宁——蓬溪快捷通道建设，建成遂宁——大英快捷通道、遂西高速公路市城区连接线，推进市县各产城组团有机链接。建成云平台、地理空间信息系统等智慧城市运用项目。抓好12个"百镇建设行动"试点镇和20个绿色小城镇试点镇建设，推进640个幸福美丽新村建设。升级改造农村电网。加大通村公路建设和管护力度，支持发展农村客货运输。继续实施城乡建设用地增减挂钩项目。

　　着力县域经济协调发展。鼓励各县（区）发挥比较优势，形成特色明显、区域互补、协调联动的发展格局。支持船山区重点发展现代服务业和先进制造业等产业；支持安居区壮大提升天然气、装备制造产业，加快融城发展；支持射洪县以西部国际技术合作产业园为重点，大力发展创新型产业；支持蓬溪县全域建设国家现代农业示范区，发展现代农业、食品加工等特色产业；支持大英县加快工业转型升级步伐，壮大提升文化旅游产业；支持开发区壮大电子产业集群，建设西部高端电子产业配套基地；支持河东新区优先发展休闲旅游、商贸会展、健康养老、职业教育等产业，打造现代服务业高地。

　　四、把开放发展作为重要抓手，融入合作共赢大格局

　　坚持向引资引企要活力。深化高位招商、高端招商、高频招商，力争招引亿元以上项目100个，到位资金420亿，实际利用外资5500万美元。积极参与中外知名企业四川行、西博会、科博会、酒博会、农博会、川商返乡发展大会等省级重大招商活动；主动对接全省电子信息、汽车制造等重点产业专题招商活动，跟进成长型产业目标企业，积极引进电子产业终端项目、新能源汽车核心部件制造等重大产业项目。务实举办泛成渝商会合作峰会、首届遂商大会和中德中欧、港澳台、长三角、珠三角、闽三角、重庆等专题投资促进活动。启动建设遂宁中德中欧产业园。坚持投资强度和亩均税收等招商项目评审制，加强招商项目落地督查，提高项目履约率和资金到位率。

　　坚持向区域合作要市场。主动融入"一带一路"、长江经济带和成渝经济区发展战略，积极对接全省"251三年行动计划"，鼓励企业参加"千企行丝路"活动，深度拓展区域经贸交流，实现进出口总额6.6亿美元。加快区域合作通道建设，推动遂宁至德阳、三台经大英至乐至、南充经蓬溪至潼南高速公路建设和成南高速扩容改造，推进成遂南达万高铁、绵遂内城际铁路和市城区至潼南界30公里Ⅳ级航道等重点项目前期工作；开展"物流通道建设年行动"，积极推进川贵广—南亚国际物流大通道建设，打造"广货北上、川货南下"的重要连接点，启动与宁波、西安等市的物流通道建设。加快申建综合保税区和国际陆港城市，推进检验检疫区域一体化和直通直放。

　　坚持向政务环境要优势。完善一窗受理、导办代办、并联审批、重大项目VIP服务等机制，打造企业和群众办事"无障碍通道"。严格按照公共资源交易目录，将政府购买中介服务、特许经营权等纳入交易平台，完善电子竞价等机制。深入推进小微企业信用体系试验区建设，开展金融守信企业红名单评选，推动银税合作至县（区），持续优化地区金融服务环境。继续办好"12345"政府服务热线。

　　五、把共享发展作为最终目的，增强人民群众获得感

　　倾力办好民生实事。始终把改善民生作为最大责任，让发展更有温度、让幸福更有质感，确保民生支出占地方一般公共预算支出的69%左右。坚持就业优先战略，加强人力资源供求信息对接服务，加大对灵活就业、新就业形态的支持力度，持续提高城乡居民收入。全面落实农民增收县（区）委书记、县（区）长负责制。实施好10项民生工程，突出办好20件民生大事，包括改善农村通村公路；推进农村渡口"渡改桥"；改造农村危旧房；改造城市危旧房棚户区；筹集公共租赁住房；提高城乡居民基本医疗保障水平；加强重大传染病和地方病防治；向年收入低于国定扶贫标准的农村低保对象发放特殊生活补贴；向持有第二代《残疾证》的低保对象发放困难残疾人生活补贴；提高公办普通高中生均公用经费保障水平；免收建档立卡贫困家庭子女幼儿园保教费；对建档立卡贫困家庭学生实行特别资助；实现103个贫困村脱贫；治理地质灾害隐患点，做好避险搬迁安置；完善村庄生活垃圾收集设施，配备转运设备；加快4个小农水重点县项目建设；实施"城市一卡通"工程，试点发放遂州通市民卡；推进"三无院落"改造工程；建设"天网工程"和互联网交通安全综合服务平台；增设遂宁往返成都始发动车。

　　推进精准脱贫攻坚。实施好10个专项方案年度工作计划，实现3.6万人脱贫。坚持小片区连片开发整村推进与精准扶持到户"双轮驱动"，抓好基础设施、医疗卫生、易地搬迁等十大扶贫工程，推进旅游扶贫、电商扶贫、科技扶贫、文化扶贫。创新扶贫投入机制，引导更多金融资金和社会资本参与扶贫开发。加大扶贫资金统筹整合和监管力度，保障扶贫重点项目投入。强化脱贫攻坚责任机制，落实扶贫目标、任务、资金、权责"四到县"制度。

　　提高公共服务水平。加快教育综合改革，优化城乡教育资源配置，全面提升教育质量。实施薄弱学校改造工程和义务教育学校标准化建设达标工程，促进义务教育均衡发展。创新推动科教园区建设，发展技术技能型高等教育，培育产学研一体研创机构。加强公共卫生工作，深化分级诊疗等3项改革试点，推动县（区）卫生应急规范化工作全覆盖，规范居民医保报付行为。落实全面两孩政策。实施全民参保计划，推进五险统一征收和稽核，加快机关事业单位养老保险制度改革。依法做好征地拆迁安置，妥善解决失地农民参加养老保险遗留问题。统筹社会救助，提高城乡低保水平，发展慈善事业，深化残疾人"量服"工作。落实全民健身战略，建设城市社区"15分钟健身圈"，扩大农村体育健身工程覆盖率。推动基层综合性文化服务中心及市图书馆、文化馆、博物馆、科技馆、规划馆和数字档案馆建设。落实公交优先发展政策，加强城市公共交通基础设施建设。

　　依法加强社会治理。深入开展信访"三无"创建活动，大力化解信访积案。健全网格化服务管理体系，加强社区、社会组织、社会工作者"三社联动"，支持工会、共青团、妇联等群团组织参与社会治理。深化城市管理体制改革，促进城市管理重心下移。健全立体化社会治安防控体系，继续推进农村"雪亮工程"，严厉打击违法犯罪活动。扎实做好安全生产工作，健全安监与监察、司法联动机制。加快市食品药品检验检测中心建设，深入开展药品器械流通领域专项整治，强化食品批发户、餐饮店、大型超市、学校周边小食摊等重点领域监管，保障群众"舌尖上的安全"。完善农民工欠薪治理体系，维护农民工合法权益。加强民间融资风险防控和化解，依法开展"打非治赖"行动。加强应急管理，提升应急处突能力。启动"七五"普法。

　　加强政府自身建设

　　2015年，我们按照市委依法治市部署，深化法治政府建设。坚持在法治的轨道上用权，完成政府机构改革，推进规范行政执法自由裁量权、解决依法行政突出问题等工作，成为全省首批具有立法资格的市。坚持在监督的环境里工作，全面落实"十项制度"，推行政府权力清单、责任清单，人大代表建议、政协委员提案办理满意率达99.6%。坚持在清新的风气中生活，自觉践行党的群众路线，落实转作风系列规定，扎实开展"三严三实"专题教育，深入整改突出问题，"三公"经费、政府文会数量持续下降。

　　2016年，我们将着力建设法治型、创新型、廉洁型和服务型政府，增强政府公信力。

　　一、坚持依法用权，建设法治型政府

　　科学民主决好策。将依法依规、阳光透明、集体决策作为行政决策的核心要件，完善重大决策合法性审查和政府法律顾问制，建立行政决策终身责任追究制度及责任倒查机制。推动地方立法，加强规范性文件备案审查。

　　公正文明执好法。规范行政执法程序，解决一批依法行政突出问题。大力推行综合执法，减少市、县（区）两级政府执法队伍种类。加强执法队伍建设，提高执法能力和水平。

　　接受监督履好职。自觉接受人大及其常委会的法律监督、工作监督和市政协的民主监督，认真听取民主党派、工商联、无党派人士和人民团体的意见。强化行政监察和审计监督，深化政务公开，主动接受公众和舆论监督。

　　二、坚持开拓进取，建设创新型政府

　　简政放权转职能。强化政府公共服务、市场监管、社会管理、环境保护等职责。按照"精简、统一、效能"的原则，推进行政体制改革和事业单位分类改革，推动扩权强镇改革。

　　创新服务提质量。提高政府公共服务效能，推动公共资源更多地向民生倾斜。积极创新服务方式，将第三方可提供的事务性管理服务主动交给市场。

　　强化执行重落实。完善大督查机制，健全能定责、可追责的考核机制。开展经济发展软环境专项整治，坚决纠正不作为、乱作为现象，坚决克服懒政、怠政的问题，打通抓落实的"最后一公里"，力破"中梗阻"。

　　三、坚持遵规守纪，建设廉洁型政府

　　拧紧思想"总开关"。切实加强政府系统党建工作，巩固拓展党的群众路线教育实践活动和"三严三实"专题教育成果，扎实开展"两学一做"学习教育。

　　扎紧制度"铁笼子"。坚持把纪律规矩挺在前面，扛稳把牢党风廉政建设主体责任。严格落实"十项制度"和"三大清单"制度，加强惩防体系建设，严肃查办腐败案件。

　　管好政府"钱袋子"。坚持厉行节约，将政府收支全面纳入预算管理并公开，严控"三公经费"支出，把有限资金用到惠民生促发展上。

　　四、坚持勤政务实，建设服务型政府

　　恪守为民之责。把人民群众对美好生活的向往作为施政方向，坚持政策向民生聚焦，服务向民生覆盖，把温暖留给群众，把实惠留给群众，把方便留给群众。

　　树立担当之志。善于攻坚克难，在难题面前敢闯敢试、敢为人先，在矛盾面前敢抓敢管、敢于碰硬，在风险面前敢作敢为、敢担责任。

　　弘扬实干之风。坚持倡实治虚、崇尚实干，不搞形象工程、政绩工程，多做打基础利长远的事。大力精减文会，做到文不过夜、事不隔天、会不开长。

　　各位代表，新起点孕育新希望，新征程谱写新篇章。让我们在省委、省政府和市委的坚强领导下，群策群力、砥砺奋进，为"决战决胜全面小康，建设绿色经济强市"努力奋斗！
